# Research progress on extraction technology and biomedical function of natural sugar substitutes

**DOI:** 10.3389/fnut.2022.952147

**Published:** 2022-08-03

**Authors:** Pengyu Lei, Haojie Chen, Jiahui Ma, Yimen Fang, Linkai Qu, Qinsi Yang, Bo Peng, Xingxing Zhang, Libo Jin, Da Sun

**Affiliations:** ^1^Institute of Life Sciences & Biomedical Collaborative Innovation Center of Zhejiang Province, Wenzhou University, Wenzhou, China; ^2^College of Life Sciences, Jilin Agricultural University, Changchun, China; ^3^Wenzhou Institute, University of Chinese Academy of Sciences, Wenzhou, China; ^4^Department of Endocrinology and Metabolism, The First Affiliated Hospital of Wenzhou Medical University, Wenzhou, China

**Keywords:** natural sugar substitutes, extraction technology, inflammation, obesity, diabetes

## Abstract

Improved human material living standards have resulted in a continuous increase in the rate of obesity caused by excessive sugar intake. Consequently, the number of diabetic patients has skyrocketed, not only resulting in a global health problem but also causing huge medical pressure on the government. Limiting sugar intake is a serious problem in many countries worldwide. To this end, the market for sugar substitute products, such as artificial sweeteners and natural sugar substitutes (NSS), has begun to rapidly grow. In contrast to controversial artificial sweeteners, NSS, which are linked to health concepts, have received particular attention. This review focuses on the extraction technology and biomedical function of NSS, with a view of generating insights to improve extraction for its large-scale application. Further, we highlight research progress in the use of NSS as food for special medical purpose (FSMP) for patients.

## Introduction

**Figure 1:**
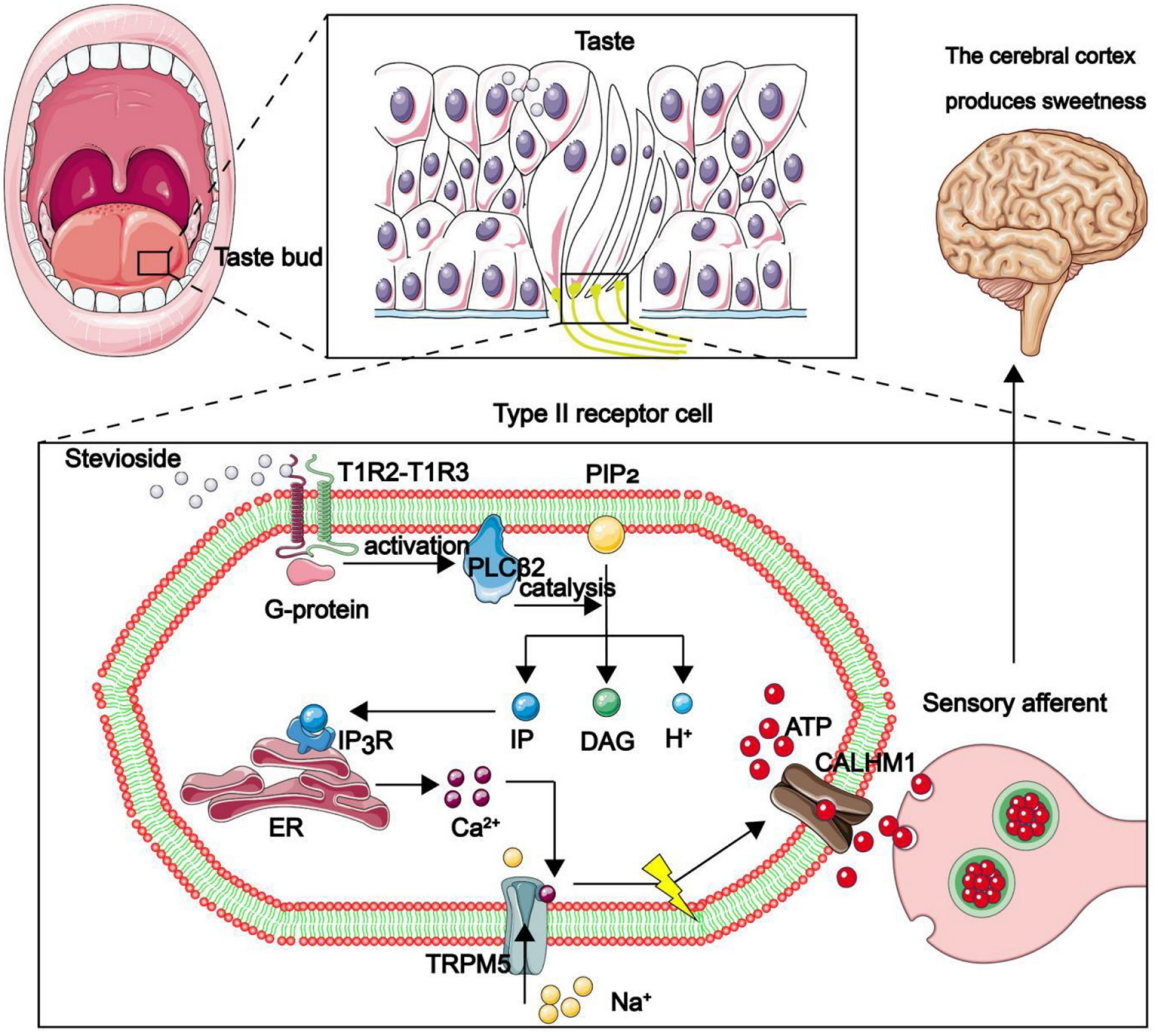
Mechanism through which the human body feels sweetness. The front end of the tongue is the main area for sweet taste. Taste pores at the front of taste buds are mainly composed of type II taste receptor cells (TRCs), while the calcium homeostasis regulatory protein 1 (CALHM1) gene is specifically expressed only in type II taste sensing cells. The sweet substance binds to the sweet receptor T1R2–T1R3 dimer on TRCs, thereby activating the coupled G protein, then the phospholipase C β 2 (PLCβ2) is deactivated. PLCβ2 acts as a catalyst, participating in the decomposition of phosphatidylinositol diphosphate (PIP2) to produce inositol triphosphate (IP3), diacylglycerol (DAG) and H ^+^. IP3 combines with inositol triphosphate receptor (IP3R) in the endoplasmic reticulum to promote release of Ca^2+^ in the endoplasmic reticulum. Ca^2+^ binds to TRPM5. The channel is opened to allow Na ^+^ to flow in, depolarize the membrane potential, promote the opening of CALHM1 channel, and the adenosine-triphosphate (ATP) outflow as a neurotransmitter transmits information to the synapse of taste afferent nerve. Finally, sweetness is produced in the cerebral cortex.

Long-term excessive intake of sucrose seriously affects human health, due to its role in development of a series of chronic diseases, such as diabetes, obesity, renal damage (1) and chronic cardiovascular diseases (2). The World Health Organization (WHO) recommends that sugar intake should not exceed 10% of the total energy intake per day (3). There are currently about 425 million diabetics in the world, a figure that is expected to reach 625 million by 2045 (4). The realization that high-sugar diet predisposes consumers to various health risks has awakened their health consciousness, thus they now actively choose the formula of sugar substitute products. Sugar substitutes, including artificial sweeteners and NSS, have replaced sugar and glucose in food. Just like sucrose, they stimulate sweet receptors in the frontal part of the tongue, thereby making the human body to feel sweet (Figure 1) (5). In addition, they are characterized by a low glycemic index (GI) and low calories.

**Figure 2:**
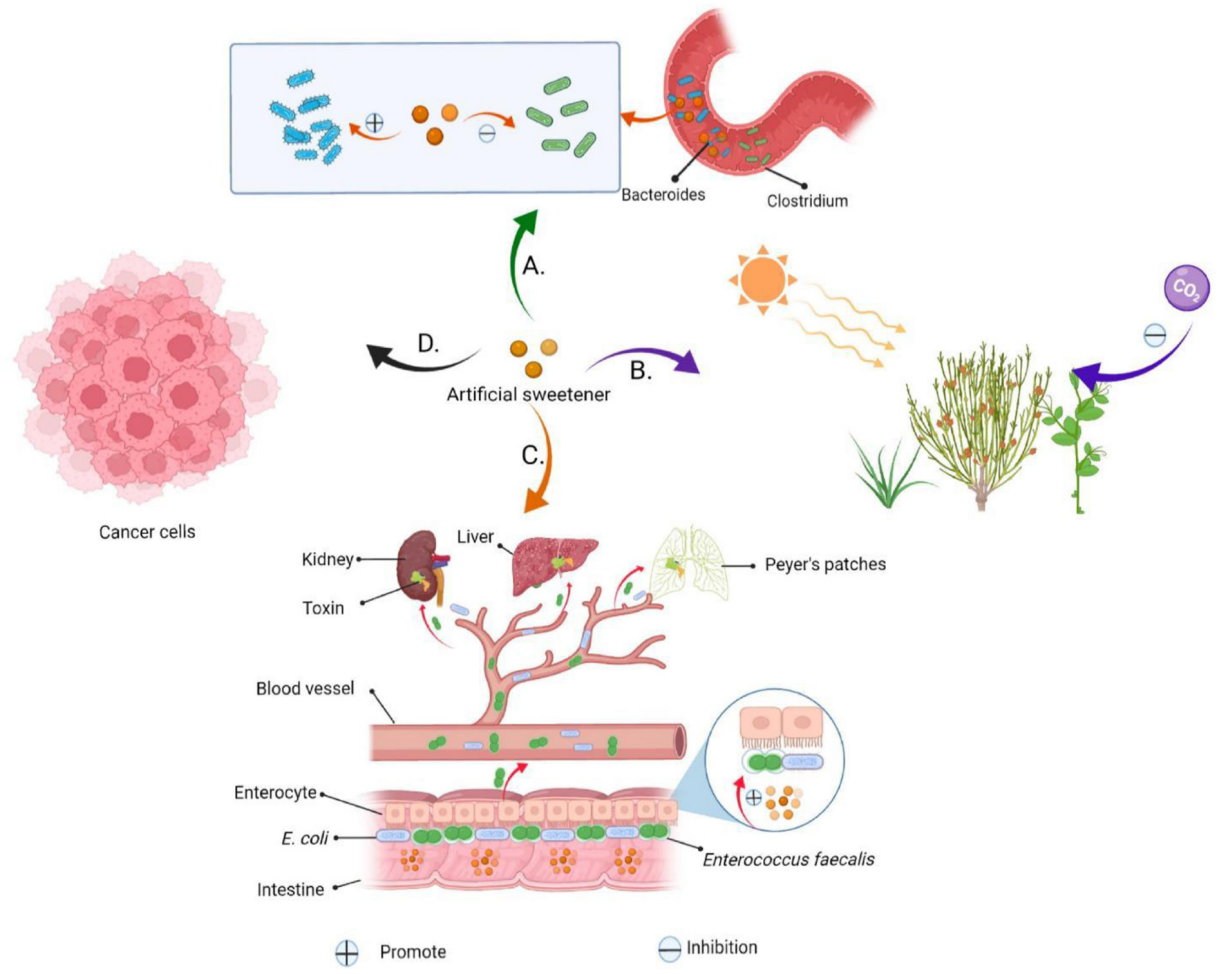
Artificial sweeteners are harmful to human health and the environment. **(A)** Artificial sweeteners mediate a reduction in glucose tolerance and insulin resistance by altering the composition of human gut microbes. **(B)** Environmental hazards caused by artificial sweeteners. **(C)** Artificial sweeteners can increase biofilm formation in *Enterococcus faecalis* and *E. coli*, adhere to and invade the epithelial cells on the intestinal wall, and kill these cells. The sweeteners can easily pass through the intestinal wall, into the blood, and accumulate in the lymph nodes, liver and spleen, thereby causing secretion of some toxins, and predisposing the body to infections, sepsis and multiple organ failure. **(D)** Artificial sweeteners increase the risk of cancer.

Common artificial sweeteners, such as saccharin, cyclamate, aspartame and sucralose, which have the advantages of high sweetness and low calorie, have been widely used as food additives for special consumer groups including diabetic and obese patients. The US Food and Drug Administration (FDA) that artificial sweeteners are safe when used within the acceptable daily intake (ADI) (6). Six artificial sweeteners have been approved by the FDA as food sweeteners (acetosulfonamide potassium, aspartame, saccharin, neotame, sucralose, and advantame) (Table 1) (12). Despite their popularity, chronic excessive consumption of artificial sweeteners may have several drawbacks: firstly, they have been shown to significantly damage the mechanism of interaction between intestinal bacteria and intestinal epithelial cells in a guasty-dependent way. This phenomenon was found to make *Escherichia coli* (*E. coli*) and *Enterococcus faecalis* harmless to the human body, even causing abdominal bloating and diarrhea, and in severe cases, sepsis and multiple organ failure (13). Secondly, studies have shown that long-term consumption of food containing artificial sweeteners affects microorganism composition in the intestinal tract, such as elevated *Bacteroidetes* and decreased number of *Clostridium*, which subsequently suppresses glucose tolerance and metabolic disorders in the long run, and even leads to development of diabetes and obesity (14). Thirdly, artificial sweeteners have a severe bitter taste, metallic taste, chemical taste and pungent taste, which is different from that of sucrose, thus does not conform to people's appealing sensory profile (15). Fourthly, consumption of artificial sweeteners has been associated with increased overall cancer risk (by 13%), including a 22% increase in breast cancer risk (16). Debras et al. (17) found that consumption of artificial sweeteners increased the risk of “obesity-related” cancers such as stomach, liver, colon and rectum by 15%. Lastly, artificial sweeteners can not only slow down the decomposition of sugar in the human body, but also interfere with the process of plant photosynthesis when discharged into the environment, which is a new type of environmental pollutant (Figure 2) (18).

**Table 1:** ADI and sweetness of artificial sweeteners.

**Artificial sweetener**	**ADI (mg/kg/BW/day)**	**Sweetness relative to sucrose**	**References**
Acesulfame potassium	15	200	(7)
Aspartame	50	160–200	(8)
Neotame	2	7,000–13,000	(6)
Saccharin	5	300	(9)
Sucralose	5	300	(10)
Advantame	0.021	37,000	(11)

**Figure 3:**
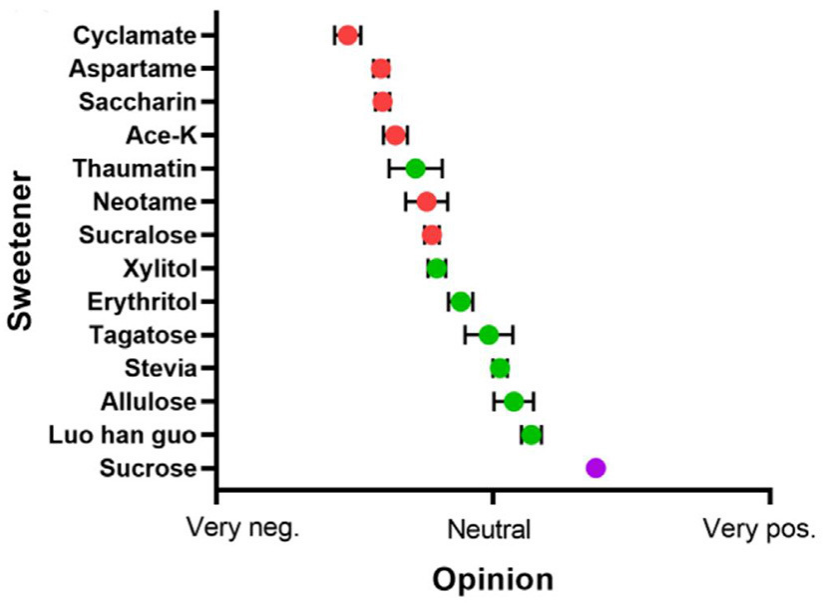
The popularity of sugar substitutes. Range from very negative (left) to positive (right), Red for artificial sweeteners, green for NSS, and purple for sucrose. Adapted with permission from (15). Copyright 2020, WILEY.

**Figure 4:**
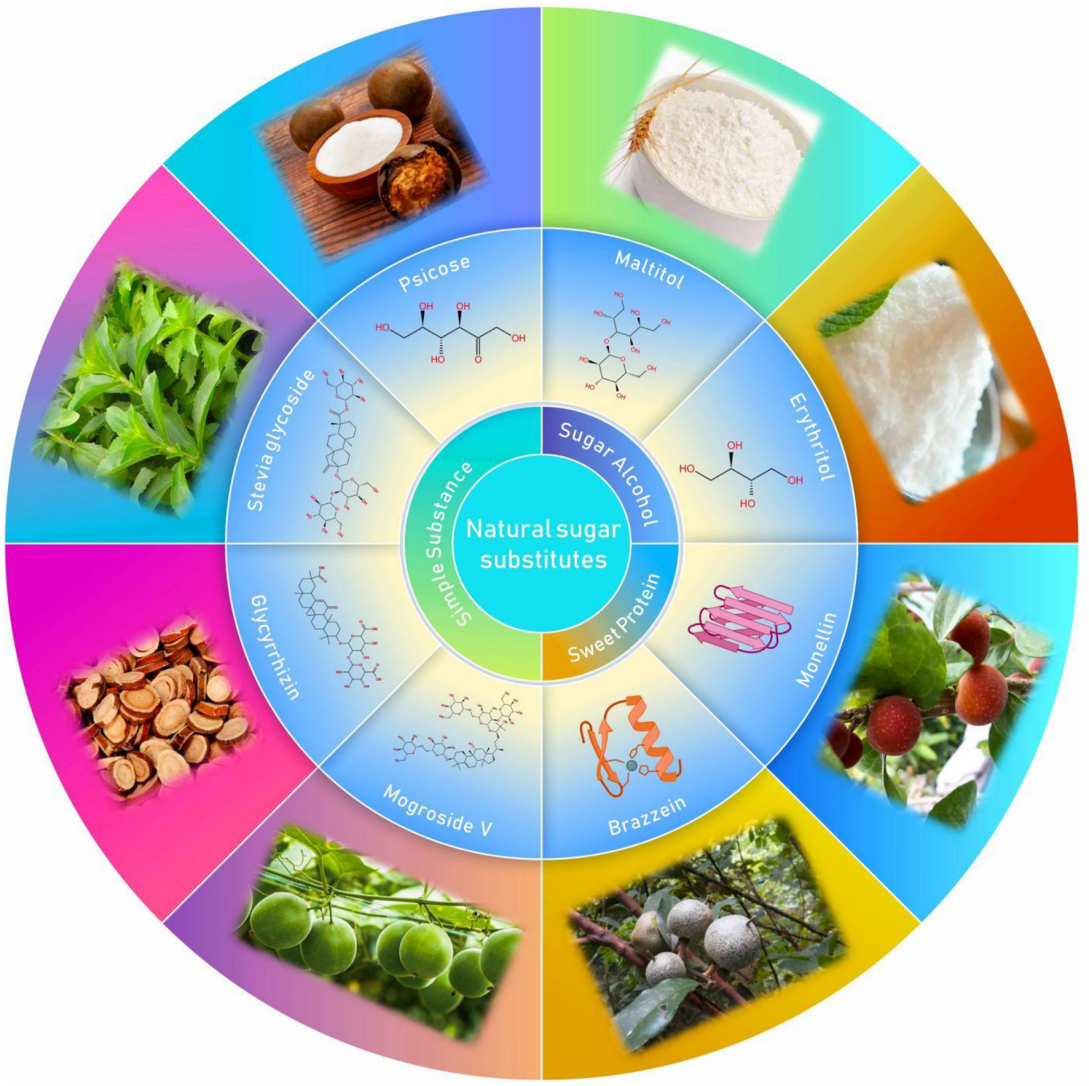
Classification, structure and source of NSS.

On the contrary, NSS are appealing to consumers because they have been associated with a healthier lifestyle and unique health care features (Figure 3) (19). In fact, these sweeteners, such as stevia glycoside, mogroside V, glycyrrhizin, psicose and erythritol (Figure 4), not only have high sweetness levels, but also have no heat, don't directly participate in body metabolism, and are associated with a certain degree of positive biomedical function, including hypoglycemic, fat accumulation inhibition, anti-inflammation and prevention of dental caries. To combat these disease, researchers in most countries have used foods to develop different regimens for patients FSMP. Part of the treatments have involved use of specially processed formula foods, which meet the special needs of nutrition or diet for people with restricted eating, digestion and absorption disorders, as well as metabolic disorders and specific diseases (20). At present, some theoretical studies and programs have been designed for clinical practice. An example of these includes providing corresponding FSMP package combinations according to patients' disease conditions and objective indicators. Although special medical food is not a drug, it can only be used as an auxiliary drug therapy. However, numerous clinical studies have shown that it can significantly reduce postoperative complications, accelerate patient recovery, and improve the quality of life during the rehabilitation process (21). In order to improve the taste and flavor of FSMP, improve the food compliance of obese, diabetic and cardiovascular disease patients and meet their need for sweet taste. Here, we review the types of processes used for NSS extraction, and highlight their advantages and limitations. In addition, we discuss the biomedical function of NSS, with a view of revealing their great potential in FSMP.

## Technologies employed in extraction of NSS

NSS, which include glycosides, sugar alcohols, ketose, sweet proteins and other bioactive substances, most of which belong to intracellular products of organisms or exist in the form of complexes, are mainly distributed in plants, microorganisms and algae. Notably, the NSS content is not only relatively low but is also difficult to directly separate and purify. The by-products of NSS can also affect the flavor and taste of the product, thus posing challenges to extraction techniques. In order to make NSS can be used in medicine, health food, cosmetics and other fields with high purity, low cost and large scale. Efficient extraction processes are imperative to quality processing of agricultural products. The currently used extraction procedures are as follows:

### Solvent extraction method

Solvent extraction method utilizes the active ingredient in different solvents with varying levels of solubility. Generally, a specific solvent is selected to dissolve as many effective components as possible. The commonly used solvents include hot and cold water, acids, alkali and organic solutions. Solvent extraction technology uses low-threshold equipment, that require simple operation processes, with a low overall cost, and allow easy industrial production (22).

The solvent extraction method is affected by various factors, key among them the crushing degree, working temperature, concentration difference and extraction time of plants. Licorice is one of the oldest and most commonly used herbs in traditional Chinese medicine, whose extract glycyrrhizin has emerged as a new natural sugar substitute (23). For example, Shabkhiz et al. (24). employed hot water extraction technique to extract glycyrrhizic acid from licorice roots, then applied the response surface method to evaluate and optimize the extraction conditions. Next, the researchers studied the effects of operating conditions on average particle size (0.5 mm) and pressure (20 bars), then separated and identified the main component of glycyrrhizic acid *via* reversed phase high performance liquid chromatography. The optimal extraction conditions were as follows: temperature 100°C, extraction time 120 min, flow rate of 15 ml/min, the amount of glycyrrhizic acid obtained reached 54.760 mg/g. Similarly, researchers have employed the solvent extraction method for traditional extraction of stevia glycoside. However, the simple solvent extraction method is associated with several drawbacks, such as complicated extraction steps, low extraction efficiency, and difficulty to remove impurities in the product, all which negatively impact the quality of the extracted product (25). In addition, stevia glycoside concentrated powder can be treated by extraction, although the recovery efficiency for stevia glycoside is low when supercritical CO_2_ is used alone. Notably, the method has a very low recovery rate of stevia glycoside when extracting fruit powder for more than 90 mins or partially purified concentrate for more than 30 mins. In view of this, solvent extraction alone cannot efficiently meet the process requirements. Previous studies have shown that with a combination of solvent extraction with other separation technologies, such as ultrasound, microwave and membrane technology, NSS can be efficiently extracted and purified from natural plants (26, 27).

### Ultrasonic extraction

This method makes use of ultrasonic cavitation to accelerate the release, diffusion and dissolution of intracellular effective substances, thus significantly improving the extraction efficiency (28). Notably, the method has various advantages, such as a short extraction time, simple operation and good repeatability, thus has been widely used for extraction of active ingredients from natural products. Apart from increasing extraction efficiency, high-power ultrasound confers additional effects, such as enzyme inhibition (29), crystallization control (30), microbial inactivation (31), as well as improved heat and mass transfer (32). Nowadays, ultrasonic technology is widely used to extract some thermally unstable active substances and foods that require low temperature processing (33).

Stevia glycoside is a new kind of natural sweetener extracted from stevia from Paraguay (34). For a long time, the solvent extraction method has been used for extraction of stevia glycosides, although the method not only produces low yields, but also the extracted products contain a lot of impurities. However, ultrasonic assisted extraction confers higher extraction efficiency, lower solvent consumption and shorter extraction time, and has also been widely regarded as an environmentally friendly extraction technology, compared to traditional methods (35). The application of ultrasonic, microwave and other emerging extraction methods for extraction of stevia glycoside has greatly improved the stevia glycoside industry. Husna et al. (35) used ultrasonic extraction method to extract stevia glycoside and reported the following optimum extraction parameters: distilled water as extraction solvent, ratio of stevia to solvent 4:30 (W/V), extraction time of 20 min, ultrasonic frequency of 20 kHz. Interestingly, the authors showed that removal of pigment impurities by SeparanAP30 and ADS-7 adsorption resin markedly improved purity of the stevia glycosides.

Previous studies have shown that solvents of deep eutectic solvent (DES), as a green alternative solvent extracted from natural active compounds, can further eliminate environmental pollution caused by ultrasonic-assisted method (36). Milani et al. (37) employed the DES-coupled ultrasonic method to extract stevia glycosides from stevia, and compared the ultrasonic-assisted extracted products with those from solvent extraction. They found that the DES-coupled ultrasonic method resulted in an almost three-fold increase in the amount of stevia glycosides relative to that obtained by solvent extraction.

Although ultrasonic extraction is a very effective method that has been used for large scale extraction of NSS, it has some shortcomings (33): failure to reach the specified ultrasonic power during the extraction process results in a very small ultrasonic effect which leads to low yield (38). In addition, it is difficult to match the power with the volume of the extraction tank. For example, when the extraction capacity is 6,000 L, the tank needs 120–180 kW ultrasonic output power, and when a high-frequency ultrasound is needed, the ultrasonic transducer cannot keep up with the capacity (39). Moreover, the equipment are costly and its routine maintenance may cause an economic burden on food manufacturers. Therefore, this method is only suitable for extracting high value-added NSS.

### Enzyme-catalyzed extraction

Enzyme catalysis has been widely used in the field of human health, agriculture, industrial production and environmental protection (40–43). Conventional extraction of substances usually requires the use of high-temperature lysis or low-temperature flash-freezing, which could reduce the activity of the extract. Enzyme catalysis allows break down of substances into small molecules at room temperature, thereby allowing generation of extracts needed for bioengineering, in an efficient and energy-saving manner. In addition, only a handful of by-products are generated, due to the specificity of the enzyme-catalyzed reaction, which is suitable for NSS with requirements of high separation and purification efficiency (44). Furthermore, the enzyme's stereoisomeric transferability can initiate an interaction with only one isomer or one conformation of the substrate, which is incomparable to general chemical catalysts (45).

Monatin [4-hydroxy-4-(3)-indolylmethyl-glutamic acid] is a sweet amino acid naturally found in the root bark of the South African plant *Scoreiton ilicifolius* (46). Studies have shown that it can be formed by tryptophan deamination to generate 3-pyruvate indole, further condensation of 3-pyruvate indole and pyruvate to form 4R-IHOG (4-(indol-3-yl-methyl)-4-hydroxy-2-oxoglutarate), and finally L-amino acid and 4R-IHOG catalyzed by L-amino acid aminotransferase to form the final target product 2S, 4R-Monatin (47). The use of monatin as a sugar substitute has potential. Meanwhile, maltitol is already widely used as a food sweetener in the market. It can be made from starch through the hydrogenation of maltose or glucose syrup. A more efficient method involves direct degradation of starch into maltose-rich products by catalytic treatment with amylases, namely β-amylase, fungal α-amylase, maltose amylase and debranching enzymes (48).

Psicose, a six-carbon rare ketose with almost zero calories, is a differential isomer that corresponds to the third carbon of D-fructose, and an ideal sugar substitute natural sugar (49). To date, nothing is known regarding chemical preparation of psicose due to the complicated product purification steps involved, as well as serious chemical contamination and production of many miscellaneous by-products (50). Nevertheless, biotransformation methods are gradually becoming the main strategy for production of psicose, due to the advantages of single reaction and simple purification steps. For example, Izumori and coworkers provided the first report of psicose extraction from *Pseudomonas cichorii*, then subsequently cloned and expressed its biosynthetic gene into E. coli JMl09. Experimental results demonstrated that under the conditions of pH 7.0, 45°C, 60% D-fructose as substrate, catalyzed by D-tagglucose 3-diisoisomerase, the binding efficiency of the enzyme can reach 90%, and the conversion of aloxone can reach 25% (51). Besides, Yoshihara et al. (52) found that *Arthrobacter globiformis* M30, which is capable of expressing C-3 differential isomerase catalyzing the conversion of D-fructose and D-psicose, exhibited high thermal resistance with an optimum temperature of 70°C. Consequently, the C-3 differential isomerase is used to catalyze conversion of D-fructose and D-psicose, to release psicose as the final product. The emergence of such enzymes, which are characterized by high thermal stability, has greatly broadened the application scenarios of enzyme-catalyzed extraction technology.

In conclusion, enzyme catalysis is suitable for the extraction of substances that are easily decomposed and unstable in nature under high temperature. Previous studies have shown that the catalytic effect of different enzymes can allow efficient extraction of different active ingredients of the substances (53). However, the enzyme-catalyzed method has some disadvantages. Firstly, the method is prone to contamination by stray bacteria owing to the fact that most enzyme catalysis reactions are under mild conditions. Secondly, enzymes are expensive and the refining process is laborious, thus the entire process of extracting them is costly. Thirdly, the process is prone to enzyme degradation in the extraction environment which leads to low extraction efficiency, due to the fact that enzymes are proteins by nature (54), The reaction conditions are therefore markedly constrained, especially when the catalytic system contains inhibitors, which will largely weaken the catalytic activity of the enzyme. Finally, at present, enzyme catalysis can only work for a limited number of compounds. Encouragingly, continuous advancements in enzyme engineering has resulted in emergence of different types of enzymes with excellent stability and high catalytic efficiency. Interestingly, the range of applicable substrates is also expanding.

### Membrane extraction method

Membrane extraction technology, which is based on selective permeability of membranes, entails the use of either natural or synthetic polymeric membranes to separate, classify, purify and enrich two- or multi-component solutes and solvents using external energy or chemical potential differences as the driving force. As a new method, this technology confers various advantages during extraction of NSS, energy saving, high single-stage separation efficiency, environmental protection, no phase change, and a simple filtration process, among others (55). At present, a variety of membrane separation and extraction processes, such as ultrafiltration (UF), nanofiltration, electrodialysis, pervaporation and gas separation have been developed (56–60). Firstly, processes for separation and purification of alkaloids are greatly constrained by various challenges, including high pollution, high energy consumption and low continuity. In the past, researchers used ceramic microfiltration membrane and organic UF membranes to separate alkaloids from broccoli and found that both membranes maintained good flux after cleaning with 1% sodium hypochlorite solution and pure water, respectively. This study provides a green method to extract alkaloids from natural products and has a good industrial application prospect (61). Secondly, numerous prebiotics fructo-oligosaccharides (FOS) exist in yacon, which not only have potential biological functions for human body, but can also be used as sugar substitutes. In a previous study, researchers concentrated and purified FOS from yacon using membrane technologies, such as UF, nanofiltration (NF) and percolation, and found that the content of talose was 50.89% while the purity of FOS was 19.75%. Therefore, UF combined with NF is a promising extraction technique, while adding filtration steps can also improve the purity of FOS (62). Thirdly, Castro et al. (25) evaluated the performance of pressure-driven membranes for the extraction of glycosides from stevia, and found that all membranes tested yielded recovery efficiencies above 90%. In addition, Montes et al. used a two-step ultrafiltration method to purify stevia glycoside from stevia aqueous extract, and found that this system effectively extracted up to 38 mg of product per 22 g of dried leaves. Therefore, membrane separation method can significantly improve the extraction rate and purity of stevia glycosides (63). At present, scholars have proved that membrane separation technology is efficient for extraction and purification of high-value molecules from natural resources. The increasing market demand for stevia glycoside also promotes the development of membrane separation (25). Fourthly, membrane separation technology has been used to extract juice from fruits and vegetables, due to its high efficiency and mild extraction environment. However, apart from destroying the sensory properties of the juice, this method also purifies the minerals, vitamins and other nutrients therein (64).

Although membrane treatment technology confers good selective filtration, it has some limitations. Firstly, starch, pectin and protein may cause serious membrane contamination, a phenomenon that is considered one of the main obstacles when using membrane separation methods for complex mixtures (57). Secondly, hydrophobic membranes can cause fouling, thereby resulting in higher maintenance costs and shorter service life. Thirdly, it is more difficult to separate isomers using this method (65). The rapid advancements in the field of materials science, have enabled optimization and upgrading of the cost and performance of membrane treatment technology. These are expected to expand the scope of application.

### Improvement of extraction methods

**Figure 5:**
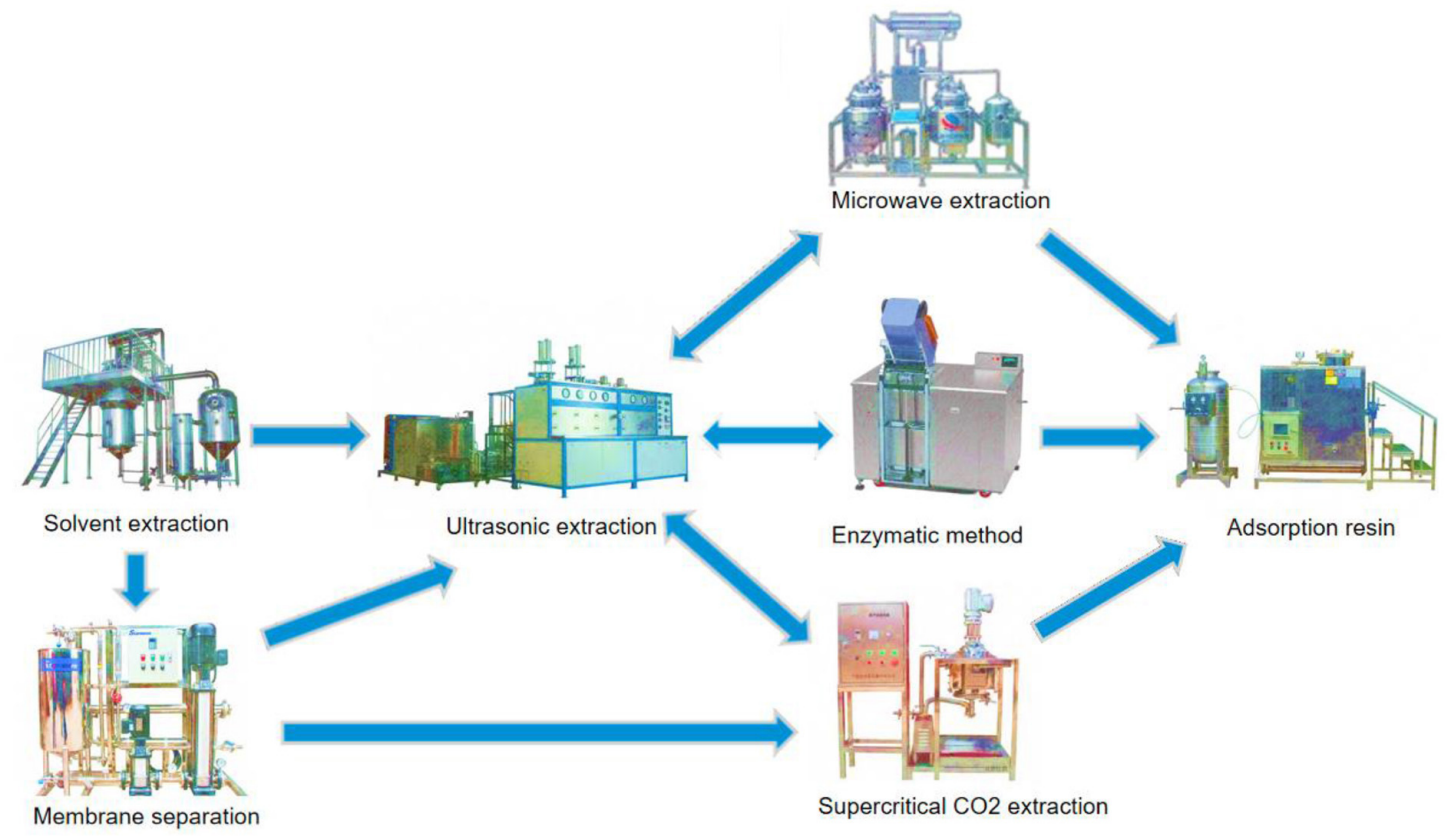
Improved schematic diagram showing the extraction method.

In view of the shortcomings associated with the above extraction methods, we can try to combine multiple extraction methods (Figure 5). Firstly, solvent extraction, ultrasonic extraction and membrane separation techniques are combined: after solvent extraction, the substance usually contains residual solvent and requires further ultrasonic treatment and membrane filtration. In addition, ultrasonic wave can effectively reduce the phenomenon of film scaling (66). Meanwhile, studies have shown that addition of supercritical CO_2_ extraction can effectively clean the organic solvent pollution on the membrane. Secondly, ultrasonic extraction can be combined with microwave co-extraction technology (67). This combination, which makes full use of cavitation effect of ultrasonic vibration and high-energy effect of the microwave, enables one to overcome the deficiency of conventional ultrasonic and microwave extraction, thus allowing for fast, efficient and reliable processing of solid samples under low temperature and atmospheric pressure (68, 69). Thirdly, ultrasonic extraction can be combined with enzyme catalysis. Notably, this combination allows for significant reduction of extraction temperature, shortens the extraction time, saves solvent, improves the yield, and does not affect structure and physicochemical properties of the extract (70). Fourthly, during enzyme catalysis, immobilized enzyme exhibits better thermal stability, operational stability and organic solvent tolerance, while macroporous resin adsorption can enhance enzyme immobilization, relative to free enzyme (71). Finally, the product obtained by supercritical CO_2_ or microwave extraction can further improve product purity through resin adsorption (72–74). Notably, simultaneous addition of ultrasonic field mediates a reduction in extraction pressure and temperature, shortens the extraction time, effectively improves the yield of NSS, and does not need any flocculant and preservative, which fully meets the production requirements of food additives (75, 76). And ultrasonic assisted method reduces resin consumption and wastewater discharge, it is also a clean and environmentally friendly extraction method (77). Therefore, a combination of ultrasonic extraction with other emerging technologies goodie a potential future research and application direction. Studies have shown that a combination with pulsed electric field, ultraviolet or infrared radiation, centrifugal distribution chromatograph and other technologies can reduce the size of equipment, speed up the start-up speed, improve the output and optimize complex process steps (78). Deepening of research, coupled with the continuous optimization and upgrading of ultrasonic combined technology, are expected to provide a variety of efficient and low-cost means for the extraction of NSS. At the same time, this combination is expected to provide a technical guarantee for large-scale industrial production of NSS, suggesting that it has a broader application in food, medicine, chemical industries.

Moreover, the current industrial production of most sugar alcohol is accomplished by chemical hydrogenation of corresponding monosaccharides and disaccharides, which has low production efficiency and is difficult to meet the needs of the expanding market (79). Nowadays, an increasing number of biotechnological fermentation methods have been investigated for the production of sugar alcohol. It can not only produce high purity NSS, such as monellin (80), thaumatin (81, 82), erythritol (83), mannitol, sorbitol and xylitol (84), but also reduce its separation difficulty and improve production efficiency, save production energy and reduce the technological difficulty of subsequent separation process. These processes are mainly achieved through biocatalysis that is aided by bacteria, fungi, yeast, as well as recombinant strains or immobilized enzyme systems. For instance, yeast can produce xylitol through the interconversion between xylose, xylitol and xylulose; Erythritol is produced by fermentation with a novel yeast, *Clavispora lusitaniae JARR-1* (85); Fructose can be converted into mannitol with 100% yield from a mixture of glucose and fructose (1:2) by heterologous fermentation of *lactic acid bacteria* (86); Sorbitol is obtained by *Zymomonas mobilis*, which converts fructose into sorbitol. In summary, biotechnological fermentation is a method of high yield, efficient production and simplified separation process (87). To date, however, fermentation processes for the remaining NSS substances have not yet been developed, mainly due to limited research system on the relevant microorganisms. Since fermentation engineering is the most rapidly developing biotechnology in the food industry, we expect that future research explorations will combine it with subsequent processing separation steps to directly generate highly pure target products. a biotechnological approach characterized with high yields, energy efficient production and simplified separation processes.

## Biomedical functions of NSS

**Figure 6:**
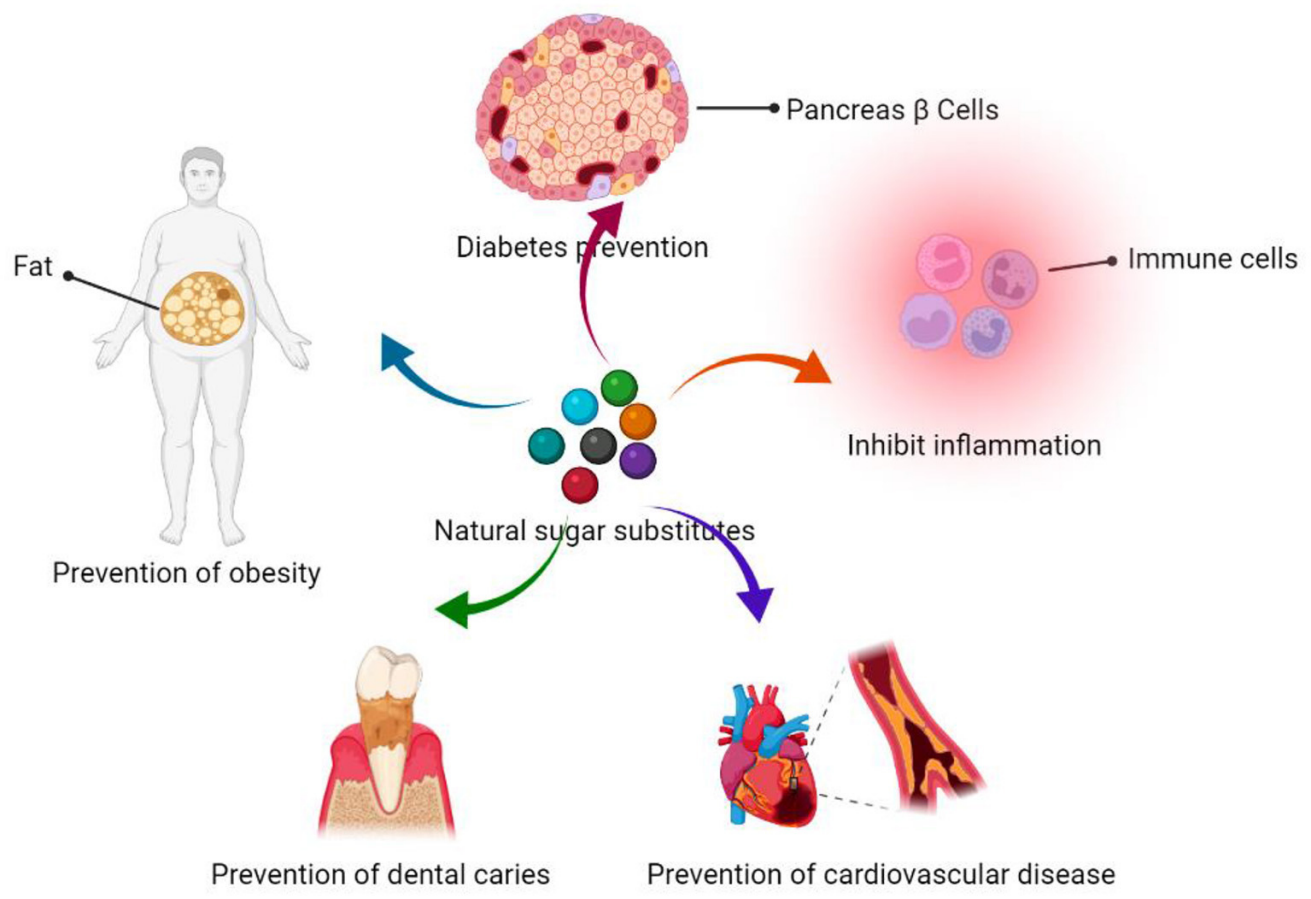
Schematic representation of the biomedical function of NSS.

NSS, as artificial sweeteners, are characterized by low GI and calories (Table 2), which make them suitable for diabetic and obese patients, as they maintain taste and flavor with minimal harm to human health. Furthermore, NSS have certain biological functions, such as prevention of diabetes and obesity, lowering blood pressure, treatment of cardiovascular diseases, anti-oxidation and prevention of dental caries (Figure 6) (89, 97, 98).

**Table 2:** Source, sweetness, heat and nature of NSS.

**Natural sugar substitutes**	**Natural source**	**Sweetness by weight**	**Caloric value (kcal/g)**	**Essence**	**References**
Sucrose (reference)	Various plants	1	3.89	Glucose	(88)
Stevia glycoside	Stevia	250–300	0	Simple substance	(89)
Mogroside V	*Momordica grosvenori*	300	0	Simple substance	(90)
Glycyrrhizin	Licorice	170	0	Simple substance	(91)
Monatin	*Scoreiton ilicifolius*	3,000	0	Simple substance	(92)
Psicose	Bioconversion	0.7	0	Simple substance	(50)
Erythritol	Yeast fermentation	0.6–0.8	0.2	Sugar alcohol	(48)
Maltitol	Starch	0.9	2.1	Sugar alcohol	(48, 93)
Sorbitol	Some fruits and vegetables	0.5–0.7	2.7	Sugar alcohol	(48)
Xylitol	Fruits, vegetables, berries, oats and mushrooms	1	2.4	Sugar alcohol	(84)
Mannitol	The leaves, stems of plants, algae, fruits	0.5–0.7	2.4	Sugar alcohol	(94)
Lactitol	A hydrogenated product of lactose	0.4	0.8	Sugar alcohol	(79)
Monellin	*Dioscoreophyllum cumminsii Diels*	3,000	Low calorie	Protein	(95)
Brazzein	*Pentadiplandra brazzeana Baillon*	2,000	Low calorie	Protein	(96)
Thaumatin	*Chlorella Neri fruit*	3,500	Low calorie	Protein	(82)

### Prevention and adjuvant treatment of diabetes

**Figure 7:**
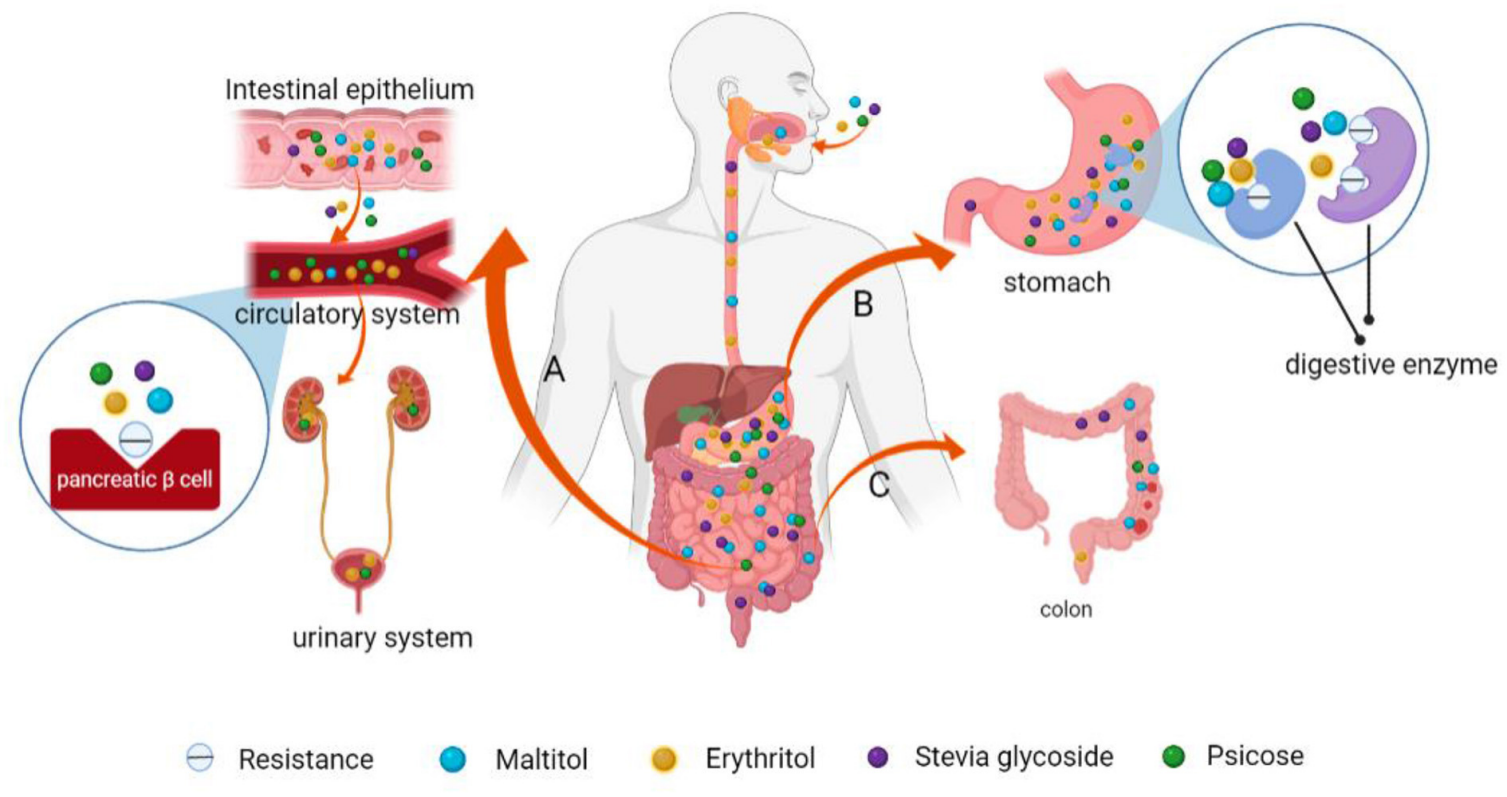
Schematic presentation of the mechanisms of low GI and low calorie levels of maltitol, psicose, stevia glycoside and erythritol. **(A)** Psicose and erythritol enter the bloodstream and are subsequently eliminated *via* urine from the body through the kidney. They do not stimulate pancreatic β-cells to produce insulin and have limited effects on blood glucose levels. **(B)** These NSS are difficult to be broken down and be absorbed by digestive enzymes in the human gastrointestinal tract. **(C)** Indigestible NSS are excreted from the intestines.

**Figure 8:**
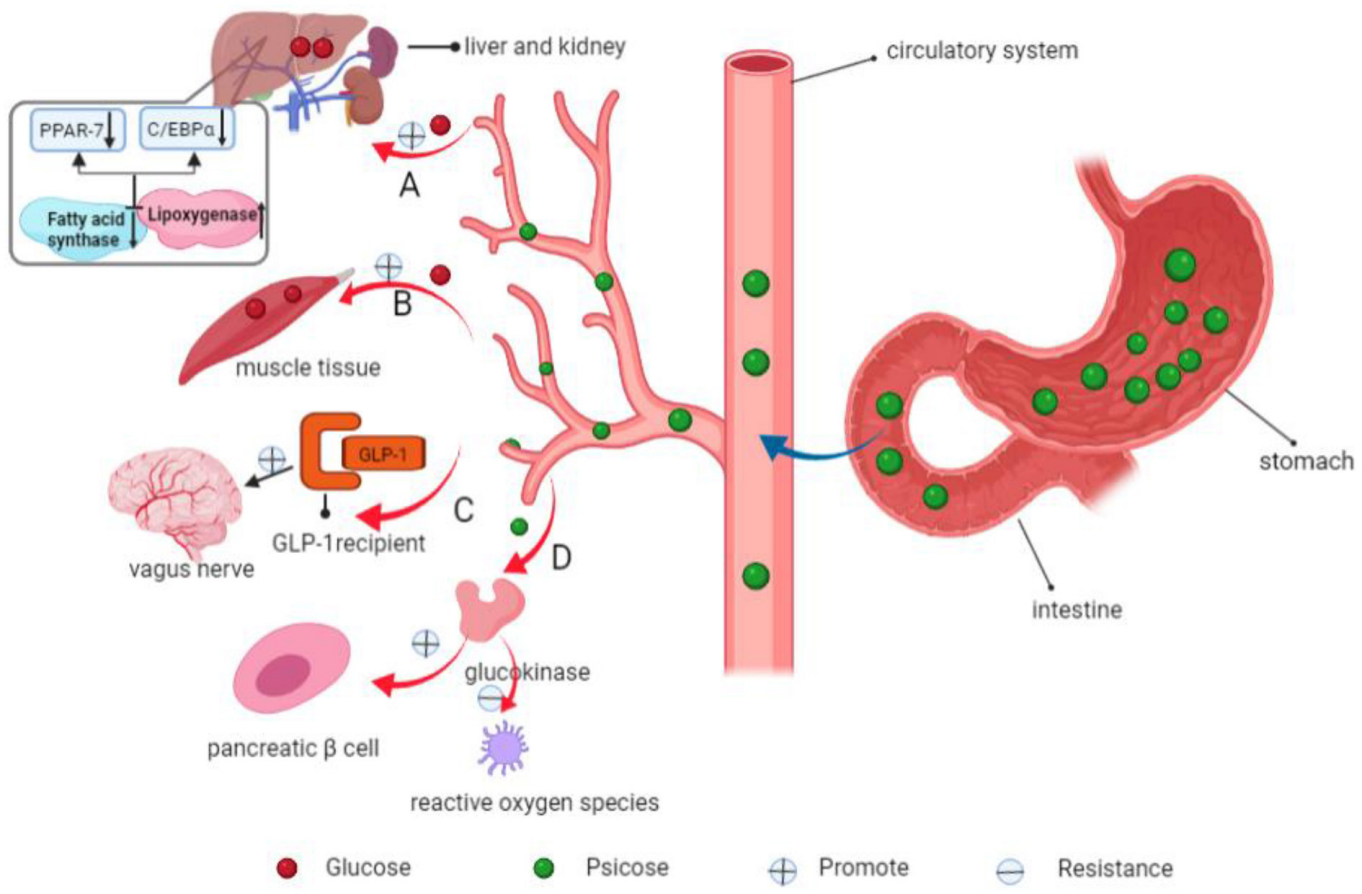
Mechanisms of psicose in decreasing blood glucose levels and inhibiting fat accumulation. **(A)** Psicose inhibits fatty acid synthase and promotes lipoxygenase activities, down-regulates the expressions of fat synthesis-related PPAR-7 and C/EBPα genes. In addition, it promotes the use of glucose in the liver and synthesis of liver glycogen. **(B)** Psicose promotes muscle glycogen synthesis. **(C)** It promotes the secretion of GLP-1 and activates the GLP-1 receptor to stimulate the vagal afferent nerve, allowing people to develop satiety. **(D)** Moreover, it stimulates glucokinase to maintain a normal GI and insulin receptor sensitivity. It scavenges for reactive oxygen species, thereby exerting antioxidant effects and plays a role in protecting pancreatic β-cells.

Diabetes is one of the most chronic diseases. It is characterized by hyperglycemia and insulin resistance, etc., accompanied by heart disease, diabetic retinopathy, kidney failure, diabetic ketoacidosis and other complications, which seriously affect the quality of life as well as physical and mental health of patients. The NSS that can be applied in FSMP for diabetic patients are classified as:

(1) Erythritol, which belongs to the polyol family, is a butanteitol prepared by microbial fermentation (99). Since the human body lacks the enzyme system to metabolize erythritol, it cannot be digested and degraded, and can only be discharged from urine through the kidney. Its metabolic pathway rarely depends on insulin, therefore, it has no influence on glucose metabolism and does not cause fluctuations in blood glucose levels (100). Bettina et al. administered erythritol solution to 12 healthy and emaciated volunteers for 4 days. They found that: erythritol significantly enhanced the secretion of human gastrointestinal hormones glucagon-like peptide-1 (GLP-1), peptide tyrosine tyrosine (PYY), and cholecystokinin (CCK), which resulted in prolonged gastric emptying time and satiety (101). Therefore, it does not cause fluctuations in blood glucose levels and has the characteristics of low GI and low calorie levels (102). Toxicological data from WHO shows that the maximum tolerable dose of erythritol is 0.669 g/kg/bodyweight (BW)/day for women and 0.89 g/kg/BW/day for men (103). In summary, because of its low GI, low calorie levels, high tolerance, unique metabolism and lack of a bitter taste after consumption (104), erythritol is a vital sweetener in foods, and has a high market value.
(2) Maltitol (1, 4-O-α-D-glucopyranose-D-sorbitol) is a nutritional sweetener (other nutritional sweeteners include sorbitol, xylitol, lacttol, and mannitol among others) (105). It is also known as hydrogenated maltitol, and its sweetness is about 90% that of sucrose (93). Since maltitol is difficult to be broken down by saliva, gastric juice, as well as pepsin among others, and only a small part of it enters the blood circulation, there is little stimulation of the insulin receptors, which does not exert marked effects on blood glucose levels (106). Maltitol has been shown to promote intestinal calcium absorption, increase bone mass and density (107). At the same time, it has a high stability, thus, it can be used as a new sugar substitute sweetener in special medicinal foods.
(3) Stevia glycoside is extracted from stevia. Its sweetness is 250–300 times that of sucrose and its calories are only 1/300 those of sucrose, thus, applications of small amounts of stevia glycoside can achieve the same taste and flavor as sucrose (89, 108). Stevia glycoside cannot be decomposed and digested by enzymes in the human gastrointestinal tract. After ingestion, it enters the colon through the stomach and small intestines, and is then fermented by intestinal microorganisms. It rarely enters the blood system, therefore, it does not increase blood glucose concentrations after ingestion. Stevia glycoside, with its properties of low calorie and high sweet degree, as well as its unique medical functions such as hypoglycemia, hypolipemia, and antibacterial, has attracted the attention of people, making it an excellent addition to food and beverage products, as well as FSMP (108). Philippaert et al. (109) fed stevia glycoside to high-fat diet-induced diabetic mice models and found that it regulated insulin secretion by enhancing the activities of Trpm5 ion channel, thereby lowering blood glucose levels. It has a certain medicinal value for obesity and diabetes (110). Other studies have found that stevia glycoside can improve glucose and insulin tolerance in type 2 diabetic rats and restore their elevated fasting glucose, serum insulin, and lipid levels to normal. Obviously, this is due to the high affinity of the stevia glycoside for insulin receptor substrate-1 (IRS-1) and glucose transporter-4 (GLUT-4), which effectively inhibits the oxidative stress of diabetic gastnemius by activating the IR/IRS-1/Akt/GLUT-4 pathway. It enhances glucose uptake and increases GLUT-4 synthesis, effectively increasing glucose uptake and oxidation in diabetic muscles. Thus, stevia glycoside can be considered as a promising plant drug for treating diabetes (111). In addition, due to its inhibitory effects on calcium ion influx, stevia glycoside can also lower blood pressure levels. Stevia glycoside has antibacterial effects and can inhibit bacterial growth when added to food, thus extending the shelf life of products (112).
(4) Psicose is a recently discovered functional monosaccharide with special health functions. Its sweetness is equivalent to 70% that of sucrose (113). It has a similar taste and flavor to sucrose and can also undergo Maillard reactions with amino acids or proteins in food (114), thus, it can be used as an ideal substitute for sucrose in food. (I) Metabolically, oral psicose is absorbed into blood through the small intestines and is then discharged by the kidneys without causing blood glucose fluctuations (Figure 7) (115); (II) Hossain et al. fed psicose to rats and found that postprandial blood glucose levels and body weights were effectively regulated. Moreover, psicose stimulated glucokinase to maintain a normal GI and insulin sensitivity (116). At the same time, it can scavenge for reactive oxygen species to exert its antioxidant effects, reducing oxidative damage to pancreatic β cells (Figure 8) (117); (III) Using healthy mice and obese diabetic mice models, Iwasaki et al. found that psicose promotes the release of GLP-1 and stimulates the GLP-1 receptor to affect the vagal afferent nerve, which enhances the development of satiety, thereby inhibiting food intake and reducing hyperglycemia (118). It can be used to meet the dietary requirements for diabetic patients.
(5) *Momordica grosvenori*, which originated from China, is a good fruit with rich nutritional values and health care functions. It has the benefits of relieving cough, removing phlegm, promoting digestion and moistening the lungs (119). The main active ingredient of this extract is mogroside V, which is about 300 times sweeter than sucrose (90). Liu et al. (120) fed rat models with 30, 75 and 150 mg/kg/BW/day of mogroside III, IV and V, respectively. They found that: mogroside V exhibited the best hypoglycemic effects. It can regulate the Phosphatidylinositol 3-kinase (PI3K)/AKT pathway, improve insulin resistance, and activate downstream AKT, thereby promoting glucose transporter 2 (GLUT2) transport, and inhibiting glycogen synthase kinase-3 (GSK-3β) activities. Thus, it increases the activities of glycogen synthase (GS), which accelerates glucose uptake by liver cells. It also has antioxidant as well as anti-hypertensive (121, 122) properties and enhances the anti-aging effects of hematopoietic stem cells (123). Its structure and properties are stable, and its flavor and sweetness are not affected by the high temperature generated by cooking and grilling, making it an ideal sweetener for FSMP with high temperature processing.
(6) Monatin, commonly known as Arruva, has two asymmetric carbon atoms, thus, it has four diastereomers. Amino et al. determined that the (2R,4R)-type is the strongest isomer with the highest sweetness levels, which is more than 3,000 times sweeter than sucrose (123), and the lowest isomer is (2S,4S)-isomer, which is 1,200–1,400 times sweeter than sucrose (124). Monatin has been shown to be safe and well tolerated in nonclinical studies, but Borje Darpo et al. R, R-monatin was found to result in a small decrease in heart rate as well as a prolonged QTcF interval that coincided with peak plasma levels in their preliminary clinical evaluation. To develop monatin as a food sweetener further, further safety assessments are needed (46).
(7) Sweet protein is a kind of natural sugar substitute with high sweetness and low calorie levels. It does not increase blood glucose levels or insulin secretion, thus, it is added to the food of diabetes and obesity patients. Currently, the known sweet proteins are brazzein, thaumatin, monellin, curculin, mabinlin, miraclin and pentadin among others (95). These proteins are isolated from plants that grow in tropical rainforests, and although they produce sweetness by stimulating the human T1R2–T1R3 sweet receptor (125), most of them have no sequence homology or structural similarities. These sweet proteins have gradually been introduced in the food industry, such as in carbonated drinks, puffed food and chocolate (95).

Monellin is a sweet protein isolated from Ceylon berry, an African shrub. Its molecular weight is about 11 kDa and it is composed of two non-covalent binding peptide chains; A and B. The A chain consists of 44 amino acid residues while the B chain consists of 50 amino acid residues. Cysteine residues in the B chain are associated with its sweet taste (126). At the same molecular concentration, its molar sweetness is about 100,000 times that of sucrose, thus, it is characterized by high sweetness and low calorie levels (127). Brazzein, a sweet protein found in red berries of the West African evergreen shrub, *Pentadiplandra brazzeana* Baillon, has a sweet taste (128). Brazzein is highly soluble and stable at a suitable pH and high temperature. Moreover, it has antioxidant, anti-inflammatory and anti-allergic properties (129). Kim et al. (96) administered brazzein or 10% sucrose of the same sweetness to mice for 15 weeks. They found that brazzein did not result in obesity and hypertrophy, neither did it disrupt glucose homeostasis or lead to insulin resistance. Although some achievements have been made in sweet protein development, its commercial costs are high: (I) Many plants with sweet protein cannot bear fruits in non-origin, which requires that production equipment must be established in origin; (II) The recombinant sweet protein produced by fermentation engineering has not reached sweet taste obtained by natural extraction; (III) For efficient and cost-effective biosynthetic systems to succeed, the product must be approved by the relevant government regulatory authorities, complete toxicology tests are required, and differences between natural extracts and biotechnological products assessed. Therefore, magnifying the unique sweet characteristics of sweet protein and developing FSMP with better tastes may be the key to successful commercialization of sweet proteins.

### Prevention and adjuvant treatment of obesity

Obesity results when the human body eats more calories than it consumes. Excess calories are stored in the body in form of fat. When the amount of fat in the human body exceeds normal physiological requirements, it slowly leads to the development of obesity. Obesity causes metabolic disorders, in which lipid metabolism is very active while glucose metabolism is suppressed (130, 131). Such metabolic changes lead to insulin resistance, hypertriglyceridemia, hypercholesterolemia and low high-density lipoprotein cholesterolemia (132). In addition, obesity can lead to heart hypertrophy and elevated blood lipid levels, which may cause a series of cardiovascular diseases (133). Some NSS inhibit fat accumulation and prevent obesity. (I) Stevia glycoside inhibits hydroxymethylglutaryl CoA reductase (HMGCR), thereby reducing low-density lipoprotein (LDL) levels in blood and preventing atherosclerosis (134); (II) By fed 5% psicose to diabetic mice models and compared their outcomes to those fed with sucrose. They found that: body weights and fat contents of mice were significantly decreased; expression levels of *PPAR-7* and *C/EBP*α genes, which are associated with fat synthesis were decreased; activities of FASN and lipid oxidase were down-regulated, and lipid levels in plasma and liver were suppressed (50, 135); (III) Li et al. fed mogroside V to liver steatosis mice models and found that mogroside V dose-dependently reduced the levels of total fatty acids, triglycerides and total cholesterol in the liver of mice. Moreover, it promotes lipolysis and fatty acid oxidation to treat liver steatosis (136–138); (IV) Intakes of artificial sweeteners significantly reduced the abundance of intestinal flora in mice and the levels of aromatic hydrocarbon receptor agonists, promoting systemic inflammation and fatty liver development. Another nehesperidin dihydrochalcone, which has no apparent health effects, may serve as a substitute for artificial sweeteners (139). Therefore, NSS can be used as a healthy alternative to traditional sugar substitutes without causing lipid metabolism disorders or weight gain, which has potential applications in the development of FSMP with functional weight-loss.

### Reduce inflammation

**Figure 9:**
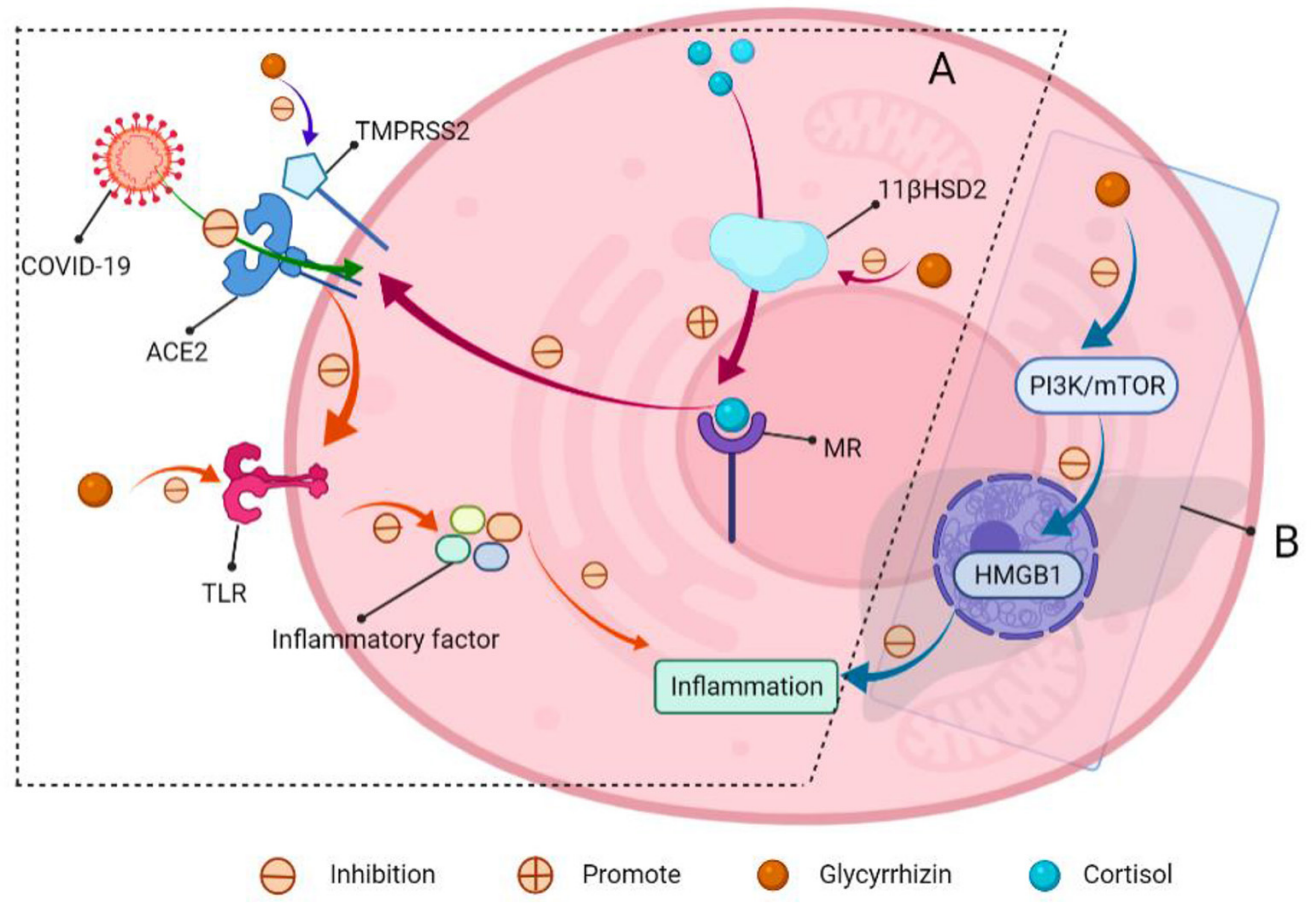
Schematic of glycyrrhizin for COVID-19 resistance and acute hepatitis therapy. **(A)** COVID-19 entry into the cells through ACE2. (I) The mineralocorticoid receptor (MR) regulates ACE2 expression, and MR activation decreases ACE2 expression. Meanwhile, glycyrrhizin promotes cortisol activation of MR by inhibiting 11-β-hydroxy steroid dehydrogenase 2 (11β-HSD2), lowering ACE2 expression and preventing virus entry into cells; (II) transmembrane protease serines TMPRSS2 (which is a cofactor that promotes viral entry into cells through ACE2). Glycyrrhizin suppressed the expression of TMPRSS2; (III) ACE2 can produce angiotensin, which inhibits TLR4 receptor expression. Meanwhile, glycyrrhizin can directly block TLR4 receptors, preventing the production of inflammatory factors and lowering lung inflammation. **(B)** Glycyrrhizin relieves acute hepatitis.

Some NSS have anti-inflammatory properties, and as a typical example, licorice is a traditional Chinese medication that has been used for centuries to treat ailments including asthma, dry cough, and other lung diseases. Glycyrrhizin is one of the key components of glycyrrhizin extract, as well as a low GI and low-calorie sweetener (140). Its sweetness is about 170 times that of sucrose, and its calorie content is nearly 0. Glycyrrhizin sweetness has a distinct woody sweetness, allowing it to be used to improve food taste and manage bitterness, as well as a sweetener in foods, beverages, confectionery, and nutritional supplements. It possesses immunomodulatory, anti-inflammatory, antiviral, neuroprotective, and antitumor properties. Compelling evidence indicates that utilizing an induced hepatitis model in mice to ameliorate liver damage, as seen by a reduction in inflammatory cytokines Institute for functional nanomaterials (IFN-γ), interleukin-6 (IL-6), interleukin-17 (IL-17), and serum alanine aminotransferase (141, 142). Other studies have shown that glycyrrhizin can inhibit inflammation and apoptosis in lipopolysaccharide (LPS) induced acute liver injury mice by inhibiting high mobility group protein B1 (HMGB1) *via* the PI3K/mTOR signaling pathway, suggesting that it could be used as a new treatment strategy for acute hepatitis (143). Furthermore, glycyrrhizin can activate aldosterone receptors in humans, lowering the expression of angiotensin-converting enzyme 2 (ACE2) (144); this plays a role in the process of corona virus disease 2019 (COVID-19) entry into human cells to prevent COVID-19 infection (145). At the same time, glycyrrhizin and its metabolite glycyrtinic acid can antagonize the Toll-like receptor 4 (TLR4) and inhibit the production of lung inflammatory factors tumor necrosis factor-α (TNF-α), IL-6, interleukin-1 (IL-1), etc., reduce lung inflammation, and reduce mortality. It can also be used as a preventive agent to lower the risk of infection and disease symptoms. Moreover, glycyrrhizin can be administered in conjunction with antiviral drugs such as chloroquine and tenofovir to protect against COVID-19 damage (Figure 9) (144).

**Figure 10:**
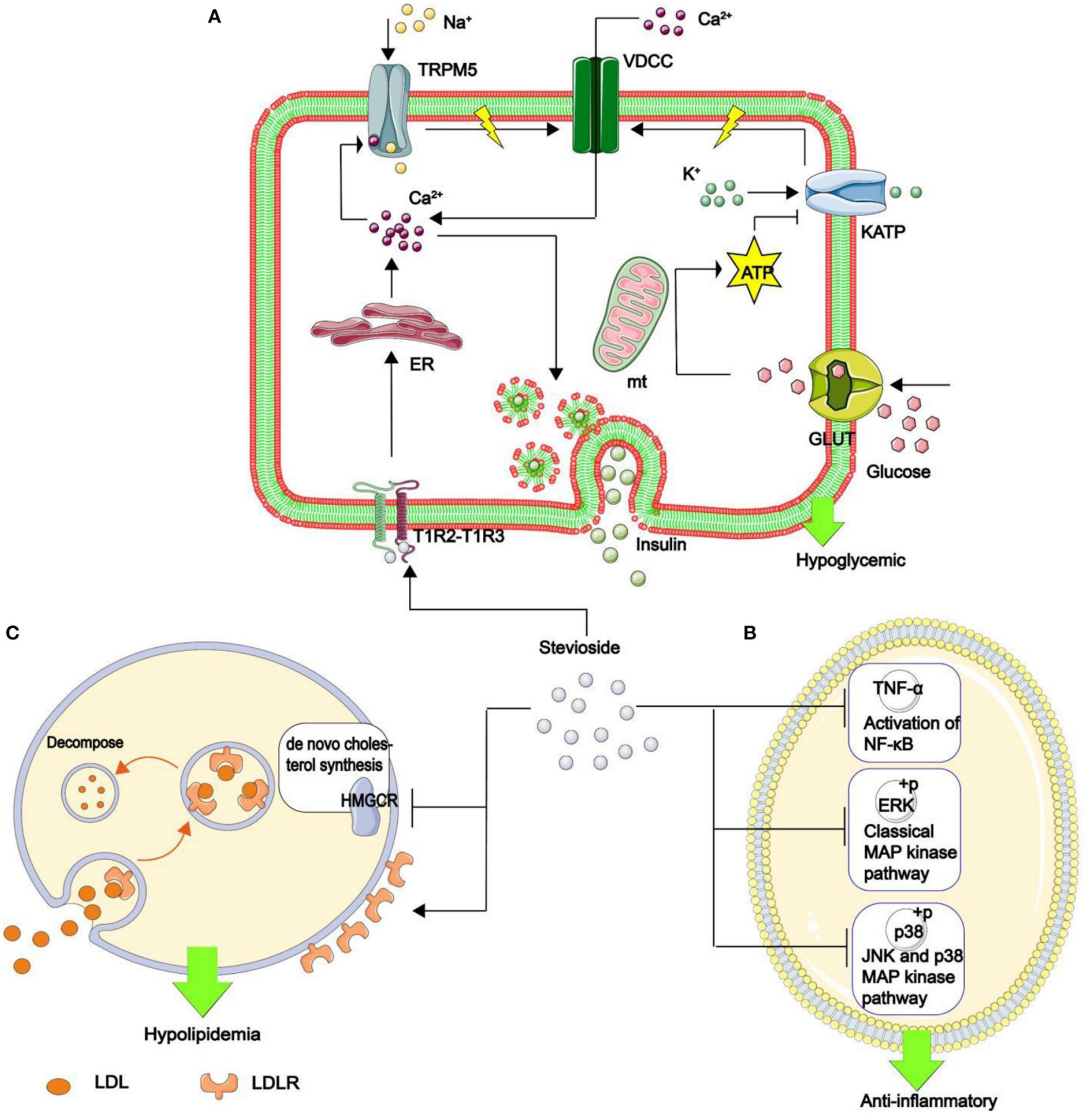
The mechanism of stevia glycoside in hypoglycemic, anti-inflammatory, and hypolipidemic. **(A)** Stevia glycoside binds to sweet receptors (T1R2–T1R3 dimer), prompting the endoplasmic reticulum to release Ca^2+^. Ca^2+^ opens the transient receptor potential ion channel protein 5 (TRPM5), allowing Na^+^ to enter and generate action potentials. At the same time, glucose is transferred to the mitochondria by enhanced diffusion of glucose transporter to generate ATP, which inhibits the opening of the ATP-sensitive potassium (KATP) and increases action potential production. Finally, the action potential opens the voltage-dependent calcium channel (VDCC), which allows Ca^2+^ to flow in. Thus, an increase in Ca^2+^ in β cells stimulates insulin secretion and hypoglycemia. **(B)** Stevia glycoside reduces the inflammatory factors IL-1β And IL-6 production, as well as the phosphorylation of two critical MAPK pathway proteins, p38 and ERK, as well as decreased TNF-α expression and hence NF-κB activation, all of which contribute to inflammation resistance. **(C)** Stevia glycoside decreases intracellular cholesterol by inhibiting HMGCR expression, the rate-limiting enzyme in the *de novo* cholesterol synthesis pathway, while increasing the cell surface LDL binding receptor (LDLR), promoting cholesterol breakdown in the cell, and the LDLR returns to the cell surface, resulting in hypolipidemia.

**Figure 11:**
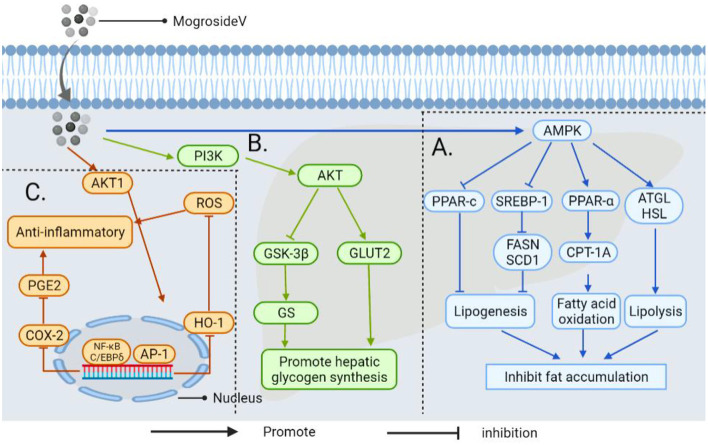
The mechanism by which androgynous mogroside V inhibits fat accumulation lowers blood glucose and exerts anti-inflammatory effects. **(A)** In LPS induced inflammatory cells, mogrosideV inhibited the phosphorylation of AKT1(protein kinase B, regulating cell proliferation and growth) and blocked AKT1 mediated NF-κB and C/EBP δ Pathway, resulting in cyclooxygenase-2 (COX-2) expression, and inhibit prostaglandin E2 (PGE2) production. In addition, dephosphorylation of AKT1 can inhibit transcriptional activator protein-1 (AP-1) to restore hemoglobin oxygenase-1 (HO-1) overexpression to the normal level and reduce reactive oxygen species (ROS) level. Thereby decreasing the occurrence of inflammation. **(B)** It upregulates phosphatidylinositol-3-kinase (PI3K) and activates downstream AKT, thereby promoting GLUT2 transport and inhibiting the activity of GSK-3β, thereby enhancing the activity of GS and accelerating glucose uptake by hepatocytes. **(C)** Mogroside V inhibits fat accumulation by activating AMP-activated protein kinase (AMPK): (I) It can down-regulate the transcription factor sterol-regulatory element binding proteins-1 (SREBP-1) and inhibit fatty acid synthase (FASN) and stearoyl-CoA desaturase 1(SCD1); (II) It can down-regulate peroxisome proliferator-activated receptor-c (PPAR-c) (involved in regulating triacylglycerol synthesis in the liver); (III) It regulates the activation of carnitine Palmitoyltransferase-1A (CPT-1A) by PPAR-α, which is involved in mitochondrial transport and oxidation of fatty acids. Thus improving liver steatosis and inhibiting fat accumulation; (IV) It enhances the expression of genes for adipose triglyceride lipase (ATGL) and hormone-sensitive lipase (HSL) in the liver to promote lipolysis.

Other NSS, such as stevia glycoside, inhibits nuclear factor kappa-B (NF-κB), a critical nuclear transcription factor implicated in inflammatory, immunological, apoptotic, and stress responses, as well as the phosphorylation of mitogen-activated protein kinase (MAPK) (Figure 10) (146). Additionally, it was found that stevia glycoside was effective in preventing ulcerative colitis caused by dextran sulfate sodium. The results showed that stevia glycoside significantly inhibited the levels of inflammatory cytokines TNF-α and IL-6 as well as the protein expression of inflammatory mediators COX-2 and NO, as well as restoring the levels of endogenous antioxidants such as superoxide dismutase, catalase and reduced glutathione in colon tissues. In mice with colitis, stevia glycoside significantly inhibited NF-κB (P65) activation by reducing P38, extracellular regulated protein kinases (ERK) protein phosphorylation (147). Li et al. (148) investigated the anti-inflammatory activity of mogroside V in RAW264.7 cells stimulated with LPS, and the results of an enzyme-linked immunosorbent assay and western blot analysis indicated that mogroside V could regulate the AKT1 pathway and inhibit inflammation (Figure 11) (149).

Diabetes, obesity, and cardiovascular disease are all associated with a series of complications, most notably inflammatory responses, that significantly impair the quality of life of patients. Of note, NSS, including stevia glycoside, mogroside V, and glycyrrhizin, have anti-inflammatory properties and may be used in FSMP to decrease the inflammatory response associated with these diseases and relieve patient discomfort.

### Prevention of dental caries

Dental caries is formed when oral bacteria (particularly *Streptococcus mutans*) utilize sucrose to synthesize insoluble glucan that adheres to the surface of teeth and forms tartar. The bacteria in tartar ferment sugar, and the sucrose molecules change a wide variety of acidic compounds. A reduction in the pH increases the concentration of hydrogen ions, which dissolves the enamel and cementum, eroding the teeth to demineralize them and subsequently destroying the organic substance to cause dental caries (150, 151). Dental caries is the most prevalent chronic disease in children, with a rising frequency among children aged 2–5 years (152). Also, NSS as a sweetener in food does not cause tooth decay. (I) Neither erythritol nor mogrosideV may be consumed or fermented by *Streptococcus mutans* and will not result in a drop in the pH on the surface of oral teeth, hence forming dental plaque (153). Additionally, erythritol may limit *Streptococcus mutans* biofilm formation, hence reducing dental caries (154, 155); (II) By having 40 caries chew maltitol gum for 2 weeks. Compared to the healthy control group, the findings demonstrated that maltitol is not an acid-generating substrate; So it will rarely lead to the synthesis of insoluble glycans by bacteria, so maltitol will not cause dental caries (156); (III) Stevia glycoside has a certain antibacterial effect and can inhibit the growth of oral bacteria Streptococcus mutans, so it can effectively prevent dental caries (157). In conclusion, this NSS may be used as sweeteners in food to help reduce dental caries in children. Additionally, due to its unique taste and anti-caries effect, NSS can be applied to toothpaste, mouthwash and other products, which can not only prevent dental caries, but also improve the taste of products and make consumers experience better.

## Challenges in the application of NSS

While NSS has a variety of beneficial biological functions for health and has a greater tolerance than synthetic sweeteners, many NSS may influence odor or sensory experience and are readily degraded or maintained for an extended period, which can be ameliorated by a combination of NSS applications. On the other hand, there are studies showing the intake of sugar alcohols could increase the number of *bifidobacteria* in their intestinal microbiomes, but no significant changes have been found in *fecal aerobic, anaerobic*, or *lactic acid bacteria*, all of which should contribute to the health of both the human body and the intestinal system (97). It has been shown that adults who consume <20 grams of sugar alcohol per day generally do not experience adverse effects on their gastrointestinal systems (158). Therefore, an appropriate amount of sugar alcohols may also have a prebiotic role in maintaining the homeostasis of intestinal microbiota. Still, the excessive long-term intake of sugar alcohols may lead to more or less intestinal diseases, depending on individual tolerance (159). This is due to the difficulty in timely digestion with large amounts of sugar alcohol, and the undigested sugar alcohol could enter the intestine as a substrate for intestinal bacterial fermentation, which produces gas that may lead to diarrhea, abdominal distension, irritable bowel syndrome, and water retention. In consequence, the food management department should develop rules for the consumption of sugar alcohol NSS in order to maximize the benefits of sugar alcohol and minimize any adverse effects on the human body. For example, the EU regulation on the supply of foodstuffs to consumers requires that products containing more than 10% sugar alcohol be clearly labeled as possibly causing gastroenteritis (79).

**Figure 12:**
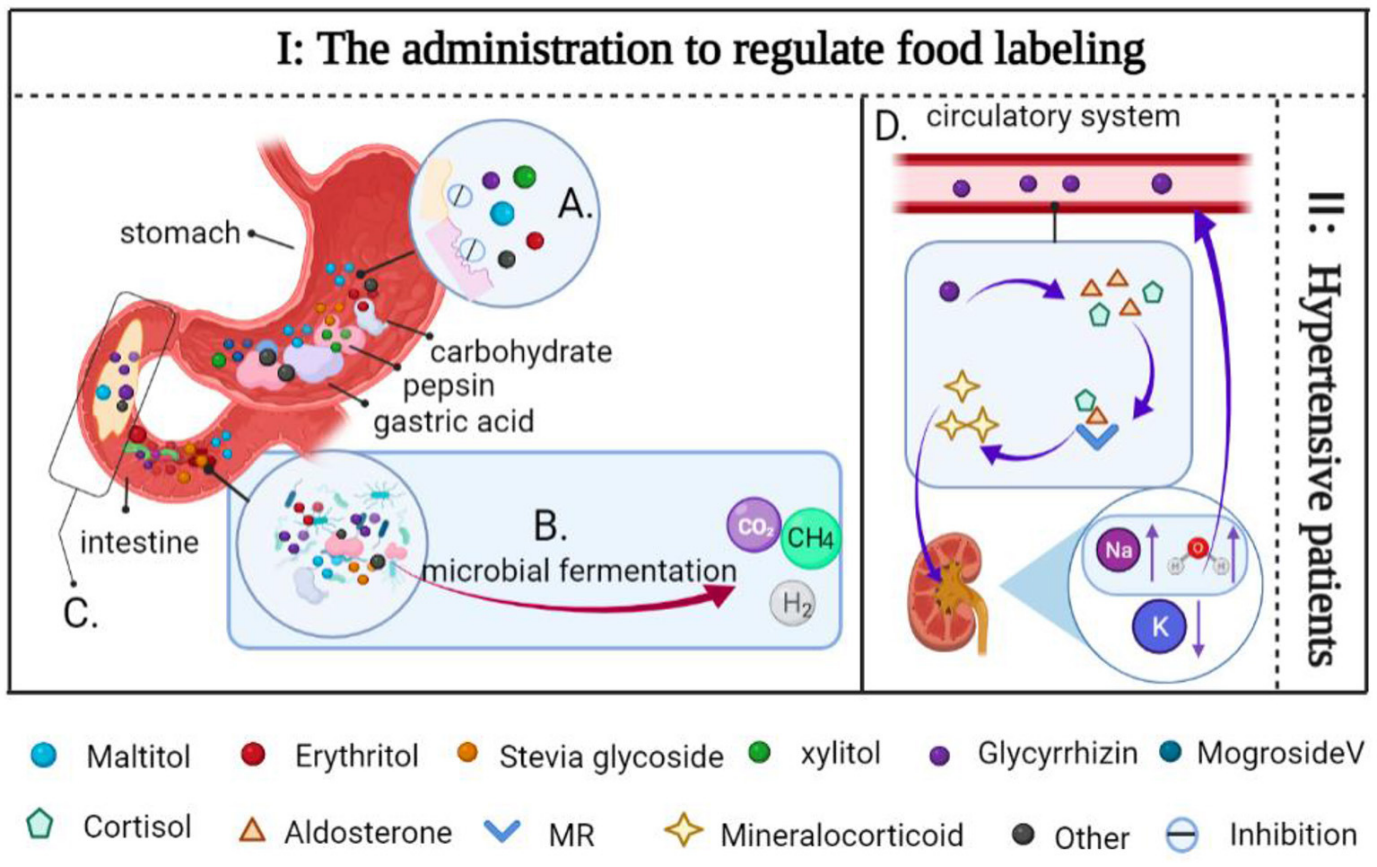
Side effects of some NSS and solutions. **(A)** Sugar alcohols are difficult for pepsin and gastric acid to digest. **(B)** Sugar alcohols and some carbohydrates are fermented together in the gut by microbes to produce gases (CO_2_, H_2_, and CH_4_). **(C)** Sugar alcohol sweeteners enter the intestine rapidly due to their inability to be digested, increased transport into the small intestine may lead to increased permeability in the lumen of the small intestine, resulting in water retention. **(D)** Glycyrrhizin inhibits the 11β-hydroxy steroid dehydrogenase activity, preventing cortisol from being converted to corticosterone, and it also increases the level of aldosterone in the human body when combined with cortisol, aldosterone, and MR, resulting in mineralocorticoid excess, increases the level of water and sodium ions in the blood, resulting in water sodium retention. As a consequence, increased blood volume results in hypertension and hypokalemia. (I) The administration to regulate food labeling to indicate the recommended intake range of NSS and the consumption group. (II) Contraindicated in patients with hypertension.

Meanwhile, the digestive system does not hydrolyze stevia glycoside (160). Due to this, they pass through the gastrointestinal tract without being absorbed and enter the colon as intact molecules (97, 161). As little as a small amount enters the circulatory system, but if consumed in excess they can also act as an energy source for the gastrointestinal microbes, altering the intestinal flora and causing discomfort (162, 163). In addition, some NSS (including stevia glycoside, glycyrrhizin, psicose, mogrosideV, etc.) with non-nutritive and low-calorie, limiting their use in FSMP to taste enhancers. Among the numerous potential adverse effects of NSS, it is critical to note that chronically high intake of licorice or its extracted complex can result in elevated aldosterone and cortisol levels in humans, thereby increasing corticosteroid production; this can cause high blood pressure (Figure 12) (164–167). Therefore, licorice and its extract must be consumed in an appropriate amount, and are contraindicated in people with hypertension. In conclusion, although excessive consumption of some NSS may cause some physical discomfort, their advantages in biomedical function are more obvious than their disadvantages. Fortunately, the adverse effects of NSS may be fully prevented with the assistance of the administration, which regulates food labeling by providing the appropriate intake range for each component and consumer group.

## Conclusion

Although the FSMPs of patients with various conditions and stages of disease vary, pleasant taste and flavor are universal criteria. With the development of laws and regulations, NSS may be extensively employed in place of conventional sugar substitutes in food, health care products, and pharmaceuticals. Therefore, we should continue to broaden the scope of natural product research in the future, screening healthy plant resources for NSS, studying the mechanism of its biomedical function, optimizing the extraction process, particularly the combination of multiple methods, and developing theoretical foundations for its application in FSMP. In conclusion, developing NSS with sensory and health attributes is the future trend.

## Author contributions

DS and LJ contributed to the conception of this review. PL, HC, and JM analyzed the literature and wrote the manuscript. YF, LQ, QY, and BP completed the figure drawings. DS, LJ, XZ, QY, and PL revised the manuscript. All authors have read and agreed to the published version of the manuscript.

## Funding

This work was supported by the National Natural Science Foundation of China (51901160), start-up funding from WIUCAS (wiucasqd2021017), and Wenzhou Science and Technology Bureau (Y2020201).

## Conflict of interest

The authors declare that the research was conducted in the absence of any commercial or financial relationships that could be construed as a potential conflict of interest.

## Publisher's note

All claims expressed in this article are solely those of the authors and do not necessarily represent those of their affiliated organizations, or those of the publisher, the editors and the reviewers. Any product that may be evaluated in this article, or claim that may be made by its manufacturer, is not guaranteed or endorsed by the publisher.

## Abbreviations

11β-HSD2: 11-β-hydroxy steroid dehydrogenase 2
4R-IHOG: 4-(indol-3-yl-methyl)-4-hydroxy-2-oxoglutarate
ACE2: Angiotensin-converting enzyme 2
ADI: Acceptable daily intake
AMPK: AMP-activated protein kinase
AP-1: Activator protein-1
ATGL: Adipose triglyceride lipase
ATP: Adenosine-triphosphate
CALHM1: Calcium homeostasis regulatory protein 1
CCK: Cholecystokinin
COVID-19: Corona virus disease 2019
COX-2: Cyclooxygenase-2
DAG: Diacylglycerol
DES: Deep eutectic solvent
*E. coli*: *Escherichia coli*
ERK: Extracellular regulated protein kinases
FASN: Fatty acid synthase
FDA: Food and drug administration
FOS: Fructo-oligosaccharides
FSMP: For special medical purpose
GI: Glycemic index
GLP-1: Glucagon-like peptide-1
GLUT2: Glucose transporter 2
GS: Glycogen synthase
GSK-3β: Glycogen synthase kinase-3
HMGB1: High mobility group protein B1
HMGCR: Hydroxymethylglutaryl CoA reductase
HO-1: Hemoglobin oxygenase-1
HSL: Hormone-sensitive lipase
IFN-γ: Institute for functional nanomaterials-γ
IL-1: Interleukin-1
IL-17: Interleukin-17
IL-6: Interleukin-6
IP3: Inositol triphosphate
IP3R: Inositol triphosphate receptor
IRS-1: Insulin receptor substrate-1
KATP: ATP-sensitive potassium
LDL: Low-density lipoprotein
LDLR: LDL binding receptor
LPS: Lipopolysaccharide
MAPK: Mitogen-activated protein kinase
MR: Mineralocorticoid receptor
NF: Nanofiltration
NF-κB: Nuclear factor kappa-B
NSS: Natural sugar substitutes
PGE2: Prostaglandin E2
PI3K: Phosphatidylinositol-3-kinase
PIP2: Phosphatidylinositol diphosphate
PLCβ2: Phospholipase C beta 2
PPAR-c: Proliferator-activated receptor-c
PYY: Peptide tyrosine tyrosine
ROS: Reactive oxygen species
SCD1: Stearoyl-CoA desaturase 1
SREBP-1: Sterol-regulatory element binding proteins-1
TLR4: Toll-like receptor 4
TNF-α: Tumor necrosis factor-α
TRCs: Taste receptor cells
TRPM5: Transient receptor potential channel protein member 5
UF: Ultrafiltration
VDCC: Voltage-dependent calcium channel
WHO: World Health Organization.
